# Evaluation of double-chambered right ventricle flow using 4D flow MRI: right ventricular helical flow may not disappear even after surgical intervention

**DOI:** 10.1093/ehjimp/qyad042

**Published:** 2023-12-12

**Authors:** Hideharu Oka, Keita Ito, Sadahiro Nakagawa, Kunihiro Iwata, Kouichi Nakau

**Affiliations:** Department of Pediatrics, Asahikawa Medical University, 2-1-1-1, Midorigaoka-Higashi, Hokkaido 078-8510, Japan; Department of Pediatrics, Asahikawa Medical University, 2-1-1-1, Midorigaoka-Higashi, Hokkaido 078-8510, Japan; Department of Medical Technology, Section of Radiological Technology, Asahikawa Medical University Hospital, Hokkaido, Japan; Department of Medical Technology, Section of Radiological Technology, Asahikawa Medical University Hospital, Hokkaido, Japan; Department of Pediatrics, Asahikawa Medical University, 2-1-1-1, Midorigaoka-Higashi, Hokkaido 078-8510, Japan

**Keywords:** double-chambered right ventricle, 4D flow MRI, helical flow

**Figure:**
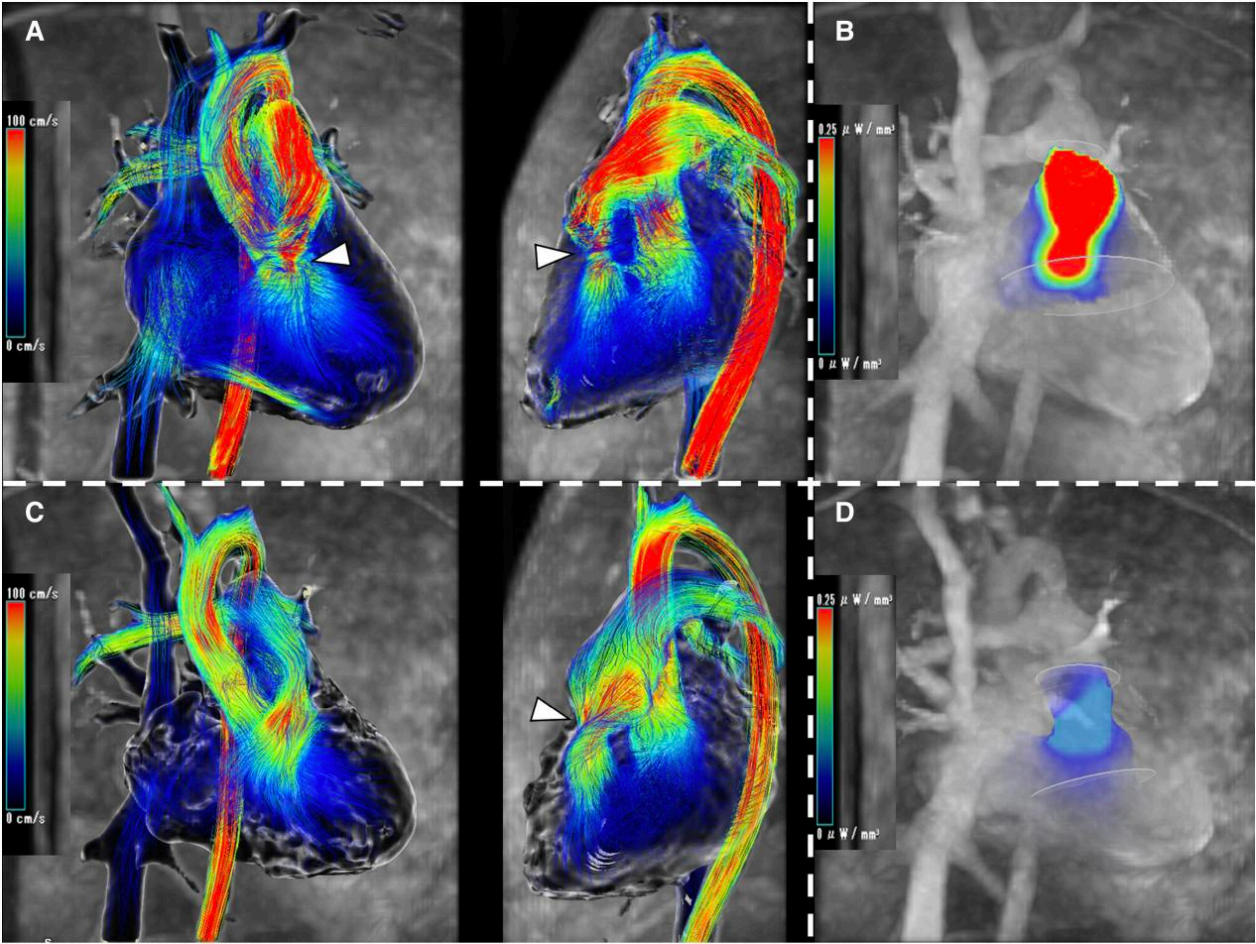


An 18-year-old male was diagnosed with a perimembranous ventricular septal defect (VSD) at birth and followed up. At 10 years old, the defect size was 3 mm, echocardiography showed right ventricular (RV) outflow obstruction (continuous wave 2 m/s), and he developed a double-chambered right ventricle (DCRV). At 18 years old, blood flow was 5 m/s by echocardiography and the pressure gradient (PG) was 37 mmHg by cardiac catheterization under sedation, and he wanted treatment. The thickened muscle bundle resection and VSD patch closure were done, and post-operative measurements with a Swan–Ganz catheter showed that the PG had disappeared. Echocardiography also showed that blood flow improved to 1.4 m/s, and the obstruction was sufficiently released. 4D flow MRI showed significant helical flow before surgery (*Panel A*, Supplementary data online, *Video S1*). The energy loss (EL, highest/mean value) was 7.54/1.87 mW (EL 0.57/0.16 mW in a healthy subject) (*Panel B*). Post-operatively, the helical flow was reduced but remained (*Panel C*, Supplementary data online, *Video S2*), and the EL was also lower at 1.45/0.42 mW, but higher than normal (*Panel D*). One month later, blood flow has accelerated to 2.3 m/s. The patient is being carefully monitored. The RV outflow obstruction is an ongoing process that may not depend only on PG, but cellular apoptosis and/or proliferation may be subjected to intracavitary blood vortices and surgery should look at imaging in pre-/post-surgical planning. In patients with DCRV, if the helical flow does not disappear after surgery, careful follow-up is needed to watch for recurrence.

## Supplementary Material

qyad042_Supplementary_Data

## Notes

**Supplementary data:** Supplementary data are available at *European Heart Journal – Imaging Methods and Practice* online.

**Acknowledgements**: We would like to thank Editage (www.editage.com) for English language editing.

**Consent**: Written informed consent was obtained from the patient for publication of this case report in line with COPE guidance.

**Funding**: Hideharu Oka has received funding from the Kawano Masanori Memorial Public Interest Incorporated Foundation for Promotion of Pediatrics/2023.

**Data availability**: The data underlying this article will be shared on reasonable request to the corresponding author.

**Author contributions**: H.O.: Conceptualization, Methodology, Validation, Formal analysis, Investigation, Writing—original draft; K.I.: Investigation; S.N.: Methodology, Writing—review; K.I.: Writing—review; K.N.: Writing—review.

## Lead author biography

Hideharu Oka graduated from Hirosaki University School of Medicine in Japan. Currently works as a paediatric cardiologist at the Department of Pediatrics, Asahikawa Medical University. His main research interests are cardiac MRI and biomarkers.

